# Origin of Locoregional Recurrences After Definitive Intensity-modulated Radiation Therapy (IMRT) for Laryngeal Cancer Determined Based on Follow-up PET/CT Imaging

**DOI:** 10.7759/cureus.3856

**Published:** 2019-01-08

**Authors:** Rafi Kabarriti, N. Patrik Brodin, Sadia Ahmed, Ivan Vogelius, Chandan Guha, Shalom Kalnicki, Wolfgang A Tomé, Madhur K Garg

**Affiliations:** 1 Radiation Oncology, Montefiore Medical Center/Albert Einstein College of Medicine, Bronx, USA; 2 Radiation Oncology, Rigshospitalet, Copenhagen, DNK

**Keywords:** laryngeal cancer, deformable registration, failure pattern, laryngeal squamous cell carcinoma (scca), positron emission tomography-computed tomography (pet/ct), intensity-modulated radiation therapy (imrt)

## Abstract

Purpose: The aim of our study was to report on patterns of failure using detailed information from follow-up positron emission tomography-computed tomography (PET/CT) scans for patients with laryngeal squamous cell carcinoma (SCCA) treated with definitive radiation therapy using intensity-modulated radiation therapy (IMRT).

Methods: One hundred and sixty-eight patients with laryngeal SCCA treated with definitive IMRT using a simultaneous integrated boost were included. The point of recurrence origin on follow-up PET/CT was determined using two separate data-driven methods. The first method, the mathematical epicenter point of origin (PO_Epi_), calculated the mathematical focal epicenter point for which the maximum distance to the surface of the surrounding volume was smaller than for any other point. The second method, maximum standardized uptake value point of origin (PO_Max_), calculated the voxel with maximum standardized uptake value (SUV) uptake within the recurrence volume. The failure pattern was then determined by whether the point of recurrence origin fell within the low, intermediate, or high-risk target volumes in the original treatment planning CT.

Results: Thirty-five primary/nodal recurrences in 33 patients were included in the analysis. In the PO_Epi_ method, 94% (33/35) of all recurrences originated either within the high-risk gross tumor volume (GTV_High-risk_) or within an average of 0.9 ± 1.3 mm from it. In the PO_Max_ method, 91% (32/35) of all recurrences originated either within the GTV_High-risk_ or within an average of 1.8 ± 1.7 mm from it. There were no recurrences outside the low-risk planning target volume (PTV_Low-risk_) for the PO_Epi_ method but there was one for the PO_Max_ method, which was 19.8 mm away from the edge of the gross tumor volume receiving 70 Gy (GTV_70_). Increasing distance between the two different origin points was strongly correlated with the size of the recurrence volume.

Conclusion: The majority of recurrences for laryngeal cancer patients treated with definitive IMRT originated from within the high-dose treatment region. This can have implications for reducing clinical target volumes while using a risk-adaptive treatment approach to both constrain dose to critical areas and further escalate the dose to the gross tumor to improve locoregional control rates.

## Introduction

Definitive radiation therapy delivered using intensity-modulated radiation therapy (IMRT) has been established as the standard of care treatment for many head and neck cancers, including laryngeal cancer, where the aim is also to preserve the larynx function [1-3]. The technical advantages of using IMRT allow for highly conformal dose-distributions and selective sparing of normal tissue structures, as well as the potential to non-uniformly escalate the dose to certain parts of the target [4-6].

Locoregional control rates for laryngeal squamous cell carcinoma treated with definitive IMRT are approximately 80% - 90% for Stage I-II [7-8] and 50% - 80% for Stage III-IV [9-12]. Previous studies have examined failure patterns for head and neck cancers in general, typically showing a higher rate of recurrence within the high-dose treatment area [1, 3, 5, 13-16].

Based on these findings, it would be intuitive to consider escalating the dose to the high-risk target volumes and taking advantage of the technical aspects of IMRT to further improve the locoregional control rates. Preliminary data shows promising results indicating that it may be safe to increase the dose to the area identified as having the highest risk of recurrence [6].

This highlights an important aspect of patterns of failure analyses to guide treatment adaptation, especially since most studies have typically just focused on determining recurrences as in, near, or out of the radiation treatment field. This has especially significant implications in head and neck radiation therapy where different dose levels are prescribed to the gross disease compared to intermediate-risk and low-risk lymph node volumes. A recent paper based on follow-up computed tomography (CT) scans of the head and neck cancer patients even showed that the results of patterns of failure analysis can depend strongly on the methodology applied to identify the site of recurrence [17].

Modern-day diagnostic imaging modalities provide added information for diagnosing locoregional failures and the ability to better determine where they originated. To this end, we present an in-depth pattern of failure analysis focused specifically on laryngeal cancer patients treated with definitive IMRT with locoregional recurrence origin determined from positron emission tomography/computed tomography (PET/CT) follow-up imaging.

## Materials and methods

Patient material and treatment information

In this IRB approved study (IRB# 2017-8424), we retrospectively identified 187 consecutive laryngeal cancer patients treated with IMRT with or without concurrent chemotherapy at our institution from 2005 to 2015. Some patients received palliative treatment, were lost to follow-up, or did not finish radiation therapy as planned, leaving 168 patients with complete information included in the analysis. Radiation therapy was delivered using simultaneous integrated boost (SIB) IMRT with 2.12 Gy/fx prescribing 69.96 Gy (~70 Gy) to the primary laryngeal gross disease and PET-positive lymph nodes (high-risk gross tumor volume: GTV_High-risk_), 59.4 Gy to the intermediate-risk lymph node regions suspect for subclinical disease (intermediate-risk clinical target volume: CTV_Intermediate-risk_), and 54.12 Gy to the negative low-risk lymph nodes (low-risk clinical target volume: CTV_Low-risk_) for the majority of Stage II-IV patients. A small number of patients with Stage I-II disease were treated with 63 - 65.25 Gy to the larynx alone. A 5 mm margin was added to the laryngeal gross tumor volume (GTV) to encompass microscopic disease and an isotropic margin of 5 mm was used to expand CTVs to planning target volumes (PTVs).

Static field IMRT using seven or nine fields was used as the treatment setup in the majority of cases, although a handful of patients were treated with volumetric modulated arc therapy (VMAT). Treatment plans were generated so that 95% of the PTV_High-risk_, PTV_Intermediate-risk_, and PTV_Low-risk_ were covered by the corresponding prescription dose unless this would lead to unacceptably high doses to organs-at-risk (OARs) according to institutional standards.

Recurrences identified on PET/CT

Electronic medical records and follow-up imaging scans were reviewed with respect to identifying any locoregional recurrences and was last updated on July 7th, 2017. Patients with recurrence identified on PET/CT imaging that was further confirmed by pathological examination were included in the detailed patterns of failure analysis.

Images were acquired using a Philips Gemini TF 16 PET/CT scanner (Philips Medical Systems, Amsterdam, Netherlands) with 512 x 512 resolution and 2.5 mm slice thickness reconstructed using a “BLOB-OS-TF” three-dimensional (3D) ordered subsets iterative reconstruction algorithm. Recurrence imaging data was exported to MIM Maestro® software, v. 6.6 (MIM Software, Cleveland, OH) prior to analysis, along with the original treatment planning CT scan and structure set.

Recurrence point-of-origin determined using two separate methods

To determine the point of recurrence origin, the PET/CT scans were co-registered with the original treatment planning CT using the MIM software’s deformable image registration framework, with a focus on accurately matching the neck region. Following image registration, MIM’s semi-automatic “PET Edge®” thresholding function was used to delineate the high uptake PET recurrence volume on the deformed PET/CT scan. The PET Edge function works through a gradient-based algorithm, detecting the steepest fall-off in standardized uptake value (SUV) values to generate contour boundaries. This method was chosen since it is less subjective and thus more reproducible compared to delineating the PET recurrence volume by hand. Furthermore, the gradient-based method in the PET Edge function utilizes relative values when determining the boundary and, as such, is not dependent on the absolute SUV values which are known to depend on the specific scanner and reconstruction protocol. 

We then proceeded to determine the point of recurrence origin using two separate data-driven methods, both implemented using Matlab, v. 2014b (The MathWorks, Inc., Natick, MA). First, we employed a previously published method that calculates the mathematical focal epicenter, defined as the point within the recurrence volume for which the maximum distance to the surface of the surrounding volume is smaller than for any other point [17]. This method, which takes advantage of the geometric information of tumor recurrences, was originally used based on recurrence volumes delineated on follow-up CT scans, whereas here, we implemented it using PET/CT-based recurrence volumes. This origin point is henceforth referred to as PO_Epi_.

Using an alternative method based on the assumption that recurrences originated from the part with the most intense PET tracer uptake, this recurrence point of origin was defined as the voxel with maximum uptake in the PET recurrence volume, henceforth referred to as PO_Max_. Using two separate methods served as a sensitivity analysis to examine whether the patterns of failure results depended strongly on the method used to determine the recurrence origin.

The derived recurrence origin points were then overlaid onto the co-registered treatment planning CT scans with the corresponding target volumes. Recurrences were scored as to whether they originated from within the different target volumes, using a Russian nesting doll principle with GTV_High-risk_, < CTV_High-risk_, < PTV_High-risk_, < PTV_Intermediate-risk_, < PTV_Low-risk_.

Statistical analysis

The number of recurrences originating from within the various target volumes was calculated separately for the PO_Epi_ and PO_Max_ methods. The percent agreement between the two methods was calculated for each target volume, along with the concordance of positively scored recurrences and Cohen’s Kappa as another measure of agreement between the PO_Epi_ and PO_Max_ methods.

The distance between the PO_Epi_ and PO_Max_ points in 3D was calculated and compared to the size of the PET recurrence volumes. We also investigated whether there was an association between recurrences being scored as originating within the smallest target volumes (GTV_High-risk_ or CTV_High-risk_) and the size of the PET recurrence volume, using the Wilcoxon rank-sum test.   

## Results

Outcomes

**Figure 1:**
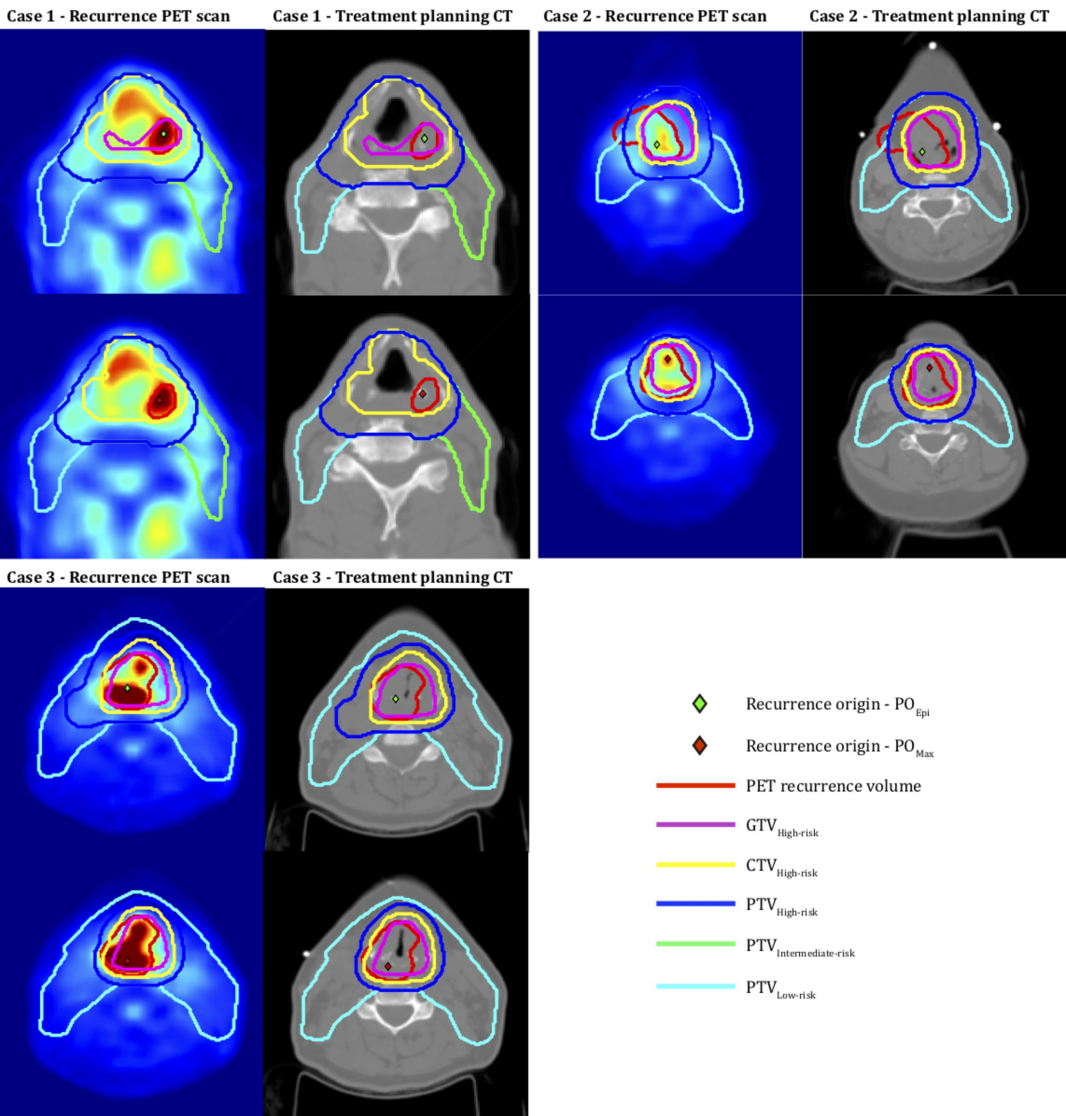
Recurrence origin points determined by the two methods for three of the patients in this analysis Calculated recurrence origin points and target volumes are shown overlayed on a cross-sectional view of the treatment planning CTs and follow-up PET scans, respectively. If the corresponding recurrence origin point falls within a given target volume, it is scored as having originated from within this and any larger target volumes encompassing that one. CT: computed tomography; CTV_High-risk_: high-risk clinical target volume; GTV_High-risk_: high-risk gross tumor volume; PET: positron emission tomography; PO_Epi_: mathematical epicenter point of origin; PO_Max_: maximum standardized uptake value point of origin; PTV_High-risk_: high-risk planning target volume; PTV_Intermediate-risk_: intermediate-risk planning target volume; PTV_Low-risk_: low-risk planning target volume

A total of 168 patients with laryngeal cancer were included in this analysis. The median interquartile range (IQR) for primary GTV volume was 33.7 cm^3^ (11.6 – 57.3 cm^3^) and 45% of these patients had Stage IVa or higher disease. With a median follow-up of 35 months, 44 patients experienced locoregional recurrence. The three-year actuarial locoregional control was 71.0%. Detailed patterns of failure were available on post-treatment PET/CT for 35 locoregional recurrences in 33 patients, of which 30 were primary and five were nodal recurrences, with more details presented in Tables 1-2. Figure 1 illustrates the point of origin determination for three patient cases using the two different methods.

**Table 1:** Target Volume in Treatment Planning Computed Tomography (CT) Detailed patterns of failure results for the 35 analyzed recurrences, with failures scored according to a “Russian doll principle” with central target volumes contained within the larger target volumes, so if a failure originated in the GTV_High-risk_, it also originated in the target volumes containing the GTV_High-risk_. CTV_High-risk_: high-risk clinical target volume; GTV_High-risk_: high-risk gross tumor volume; n: number; PO_Epi_: mathematical epicenter point of origin; PO_Max_: maximum standardized uptake value point of origin; PTV_High-risk_: high-risk planning target volume; PTV_Intermediate-risk_: intermediate-risk planning target volume; PTV_Low-risk_: low-risk planning target volume

		Target volume in treatment planning CT				
		GTV_High-risk_	CTV_High-risk_	PTV_High-risk_	PTV_Intermediate-risk_	PTV_Low-risk_
PO_Epi_ method	# of recurrences (% of total)	24 (69%)	32 (91%)	33 (94%)	34 (97%)	35 (100%)
PO_Max_ method	# of recurrences (% of total)	18 (51%)	30 (86%)	32 (91%)	33 (94%)	34 (97%)
Agreement metrics	Agreement n (%)	25 (71%)	33 (94%)	34 (97%)	34 (97%)	34 (97%)
	Concordance of positively scored recurrences (%)	67%	94%	97%	97%	97%
	Cohen’s Kappa	0.42 (max: 0.65)	0.72 (max: 0.72)	-	-	-

**Table 2:** Patient Characteristics of the Laryngeal Cancer Patients with Locoregional Recurrence Included in This Study (n = 33) *Smoking status missing for one patient n: number; SD: standard deviation; y: years

Patient Characteristics	
Median (range) follow-up (months)	31 (8 – 122)
Age (y), mean ± SD	65.1 ± 12.6
Subsite, n (%)	
Supraglottis	17 (52)
Glottis	2 (6)
Subglottis	1 (3)
> 1 subsite	13 (39)
Stage, n (%)	
I	1 (3)
II	3 (9)
III	12 (36)
IV	17 (52)
Gender, n (%)	
Male	26 (79)
Female	7 (21)
> 10 pack year smoker*, n (%)	
Yes	26 (81)
No	6 (19)

Method 1: PO_Epi_

Using the first method to calculate the mathematical focal epicenter (which is the point within the recurrence volume for which the maximum distance to the surface of the surrounding volume is smaller than for any other point), 94% (33/35) of the recurrences originated within the PTV_High-risk_, with only two recurrences occurring outside the PTV_High-risk_ but within the PTV_Intermediatde-risk_ or PTV_Low-risk_. The two failures occurring outside the PTV_High-risk_ were nodal failures, one which originated within the PTV_Intermediate-risk_ and the other within the PTV_Low-risk_. There were no recurrences scored as originating from outside the PTV_Low-risk_. The nine recurrences that originated in the PTV_High-risk_ but outside the GTV_High-risk_ were on average within 0.9 ± 1.3 mm from the edge of the GTV_High-risk_.

Method 2: PO_Max_

Using the second method to calculate the point of recurrence origin (which was defined as the voxel with maximum uptake in the PET recurrence volume), we found that 91% (32/35) of the recurrences originated within the PTV_High-risk_, with only three recurrences occurring outside the PTV_High-risk_ but within the PTV_Intermediatde-risk_ or PTV_Low-risk_. Of these three failures, two were nodal failures, one which originated within the PTV_Intermediate-risk_, and the other within the PTV_Low-risk_. The 14 recurrences that originated in the PTV_High-risk_ but outside the GTV_High-risk_ were on average within 1.8 ± 1.7 mm from the edge of the GTV_High-risk_. There was one locoregional recurrence originating outside the PTV_Low-risk_ with this method but it was within 2.5 mm from the edge of the PTV_Low-risk_ and 19.8 mm from the edge of the GTV_High-risk_.

**Figure 2:**
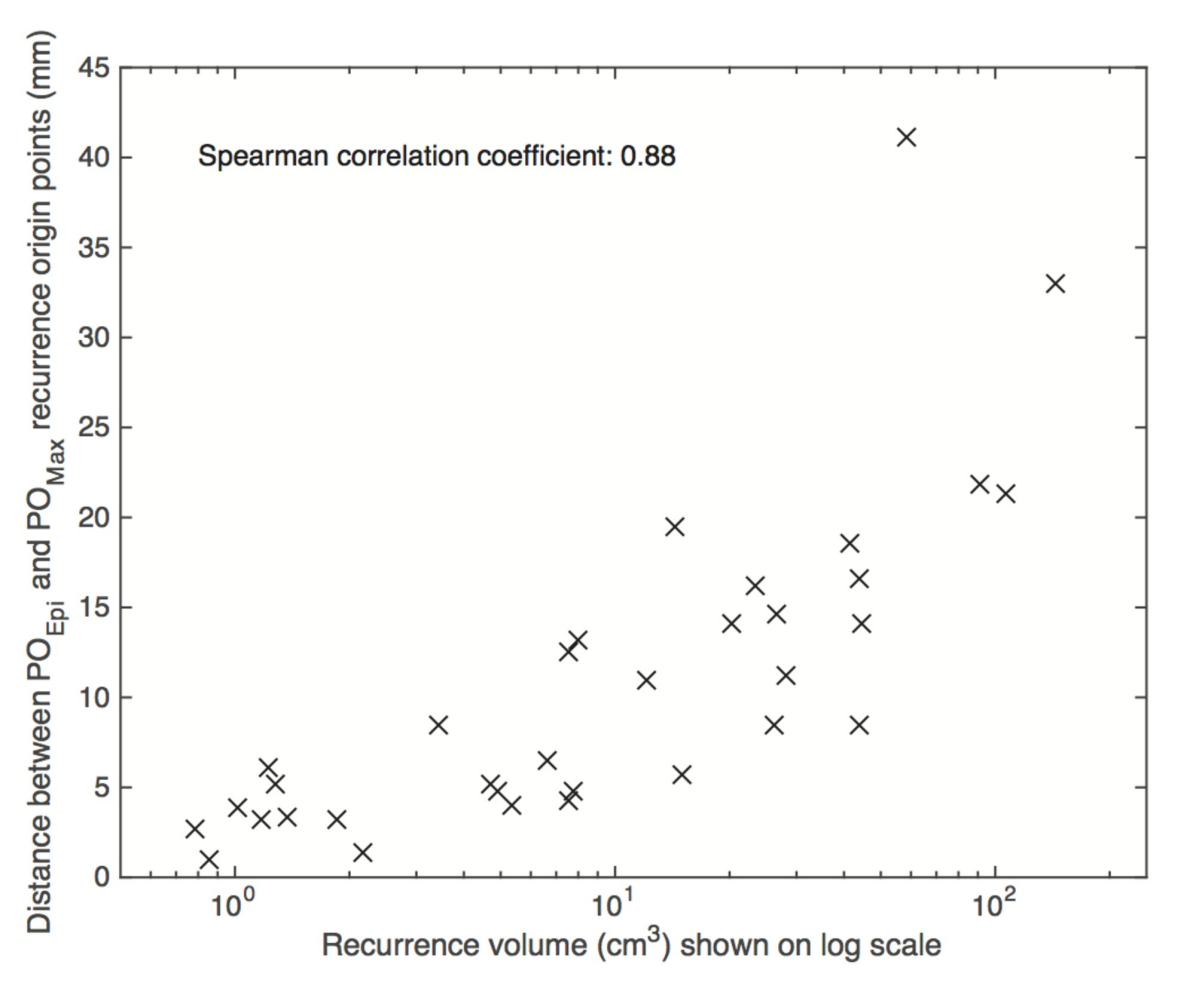
Correlation between the size of the recurrence volume and the distance between recurrence origins identified by the two methods Recurrence volume is shown on log scale for clearer visual representation. PO_Epi_: mathematical epicenter point of origin; PO_Max_: maximum standardized uptake value point of origin

There was good agreement between the two methods in classifying the target volumes from which recurrences originated with 67% concordance for GTV_High-risk_, 94% for CTV_High-risk_, and 97% for PTV_High-risk_. The 3D distance between the origin points from the two different methods was on average 11.7 ± 10.1 mm. The distance between the two different origin points was strongly correlated with the size of recurrence volume with a Spearman’s correlation coefficient of 0.88, as shown in Figure 2. Therefore, we found it pertinent to explore whether the patterns of failure results found in this analysis were dependent on the size of the PET/CT recurrence volume. However, there was no significant association between the size of the recurrence volume and whether recurrence origin points fell within the GTV_High-risk_ (p = 0.85 for Method 1, p = 0.99 for Method 2) or the CTV_High-risk_ (p = 0.28 for Method 1, p = 0.31 for Method 2).

## Discussion

Using two independent methods to analyze the origin of recurrence based on post-treatment PET using either the mathematical epicenter or max SUV value in the area of recurrence, we found that the majority of the locoregional recurrences for laryngeal cancer patients treated with definitive radiation therapy using IMRT in this analysis originated from within the high-risk treatment region with 94% for Method 1 and 91% for method originating within the PTV_High-risk_.

Although the two methods showed strong agreement, there is currently no gold standard method to truly determine the origin of recurrence, which limits our ability to determine if one method is better compared to the other. Based on the results obtained from this study, it is possible that the most reliable method for determining the point of recurrence origin may be a combination of both methods that take into account the geometric information, as well as the PET tracer uptake data of the recurrence volume. It is also important to consider the anatomical area of the larynx when determining the most appropriate method, as the applicability depends on how a recurrence is likely to expand and grow once manifested.

While we know from other studies that most recurrences in head and neck cancers occur within the high-risk regions (which is consistent with our findings), we further determined in this study whether the recurrences occurred within the GTV, CTV, or PTV high-risk regions [1, 3, 5, 13-16]. Part of this work was presented at the 58th annual American Society for Radiation Oncology 2016 meeting (Abstract: Kabarriti R, Brodin NP, Ohri N, et al.: Patterns of Failure and Origin of Recurrence on PET/CT for Laryngeal Cancer Patients Treated with Definitive IMRT, 58th Annual ASTRO Mtg., Boston, MA, Sept. 28th 2016, presentation #1135). Furthermore, the failure pattern in our study was determined through data-driven methods rather than subjective judgment, strengthening these findings. Given that the majority of recurrences occurred within the GTV_High-risk_ or within less than 2 mm from the GTV_High-risk_, one can hypothesize that escalating the dose to the gross tumor might improve local control rates. Prospective studies examining whether some patients may benefit from dose escalation to gross disease, while perhaps reducing the dose to intermediate- and low-risk lymph node regions, are warranted. It has been shown that high doses to the PET-positive GTV can be delivered safely to head and neck cancer patients undergoing definitive radiation therapy [18]. 

The limitations of this study include its retrospective nature. Additionally, fusion between follow-up PET/CT scans and treatment planning CT scans were done by using deformable image registration. Although we checked that the deformation vector fields were smooth and that no folding occurred by ensuring that the Jacobian was positive definite, it is possible that the deformation may have introduced some uncertainty as to where the origin of recurrence is precisely determined. Nevertheless, a strength of this analysis is that it was limited to a homogenous cohort of head and neck cancer patients all with laryngeal squamous cell carcinoma.

## Conclusions

In conclusion, our findings demonstrate that the majority of recurrences for this single-institution cohort of laryngeal cancer patients treated with definitive radiation therapy using IMRT originated from within the high-dose treatment region. Prospective risk-adaptive strategies exploring alternatives for selective boosting of areas that carry a high risk of recurrence are warranted.
